# Idealism versus realism: the case of overcrowding in railways amidst COVID-19 pandemic

**DOI:** 10.11604/pamj.2022.42.87.35422

**Published:** 2022-06-02

**Authors:** Shiv Hiren Joshi, Aditya Suhas Dhonde

**Affiliations:** 1 Department of Community Medicine, Jawaharlal Nehru Medical College, Datta Meghe Institute Medical Sciences (Deemed to be University), Sawangi (Meghe), Wardha, Maharashtra, India

**Keywords:** Overcrowding, COVID-19, health education

## Image in medicine

The image is a display of the stark reality of travelling in a general railway coach amidst the COVID-19 pandemic. This image was captured during a visit to Madhya Pradesh from Maharashtra five months ago. The government removed the COVID-19 restrictions from April 1^st^, 2022, which is three months after this image was taken. Overcrowding is an important public health problem which not only facilitates the disease transmission process but also expedites it. This issue gets further complicated while travelling in trains. Within the overcrowded coaches of the train, it is highly likely for the diseases to spread. While infections spreading through aerosols and droplets are a concern, especially for conditions like COVID-19, it must also be considered that the trains have multiple stops at multiple stations, which also leads to an exchange of travelers who may be infected asymptomatic as well as symptomatic patients. This pre disposes the travelers to a substantial risk of getting exposed to the pathogens due to greater opportunities for such exposure to happen. This image mocks the gap between the idealism of what should be done during the pandemic and reality. While it is the responsibility of the government to implement stringent public health interventions to prevent the spread of communicable diseases, it may be challenging to provide practical solutions to achieve a balance between idealism and realism in a developing country with such a large population. However, this image advocates the need for deliberations on how to effectively implement the public health intervention strategies.

**Figure 1:**
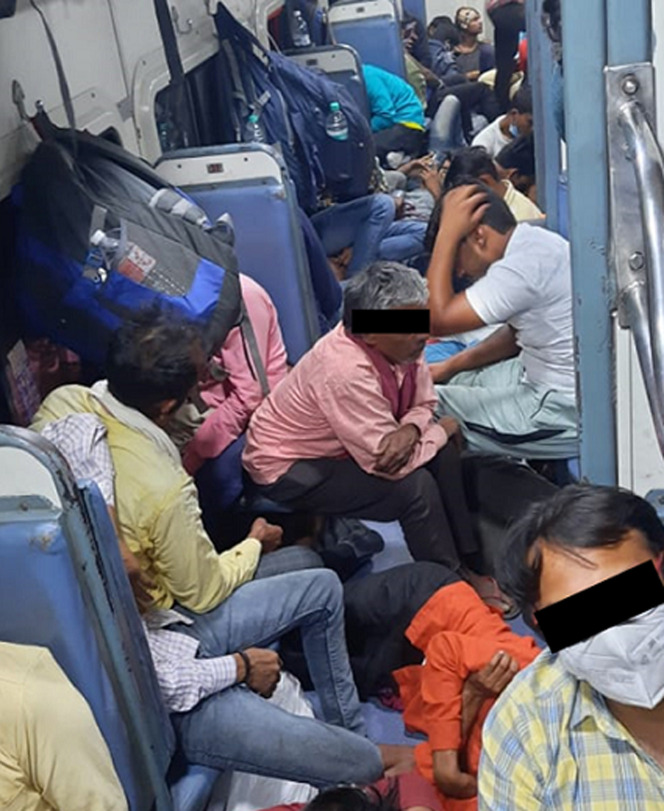
overcrowding in a general railway coach where passengers are not following COVID-19 restrictions

